# Apremilast Pharmacogenomics in Russian Patients with Moderate-to-Severe and Severe Psoriasis

**DOI:** 10.3390/jpm11010020

**Published:** 2020-12-29

**Authors:** Dmitry A. Verbenko, Arfenya E. Karamova, Olga G. Artamonova, Dmitry G. Deryabin, Alexander Rakitko, Alexandr Chernitsov, Anna Krasnenko, Artem Elmuratov, Victoria S. Solomka, Alexey A. Kubanov

**Affiliations:** 1 State Research Center of Dermatovenereology and Cosmetology, Korolenko St., 3, bldg 6, 107076 Moscow, Russia; karamova@cnikvi.ru (A.E.K.); artamonova_olga@list.ru (O.G.A.); dgderyabin@yandex.ru (D.G.D.); solomka@cnikvi.ru (V.S.S.); kubanov@list.ru (A.A.K.); 2 Genotek Ltd., Nastavnicheskiipereulok 17/1, 105120 Moscow, Russia; rakitko@gmail.com (A.R.); chernitsov@genotek.ru (A.C.); krasnenko@genotek.ru (A.K.); artem@genotek.ru (A.E.)

**Keywords:** apremilast, psoriasis, genetic risk score, single-nucleotide polymorphism, clinical outcome

## Abstract

One of the target drugs for plaque psoriasis treatment is apremilast, which is a selective phosphodiesterase 4 (PDE4) inhibitor. In this study, 34 moderate-to-severe and severe plaque psoriasis patients from Russia were treated with apremilast for 26 weeks. This allowed us to observe the effectiveness of splitting patient cohorts based on clinical outcomes, which were assessed using the Psoriasis Area Severity Index (PASI). In total, 14 patients (41%) indicated having an advanced outcome with delta PASI 75 after treatment; 20 patients indicated having moderate or no effects. Genome variability was investigated using the Illumina Infinium Global Screening Array. Genome-wide analysis revealed apremilast therapy clinical outcome associations at three compact genome regions with undefined functions situated on chromosomes 2, 4, and 5, as well as on a single single-nucleotide polymorphism (SNP) on chromosome 23. Pre-selected SNP sets were associated with psoriasis vulgaris analysis, which was used to identify four SNP-associated targeted therapy efficiencies: *IL1β* (rs1143633), *IL4* (*IL13*) (rs20541), *IL23R* (rs2201841), and *TNFα* (rs1800629) genes. Moreover, we showed that the use of the global polygenic risk score allowed for the prediction of onset psoriasis in Russians. Therefore, these results can serve as a starting point for creating a predictive model of apremilast therapy response in the targeted therapy of patients with psoriasis vulgaris.

## 1. Introduction

Psoriasis is a chronic inflammatory disease that affects 2–3% of the world’s population [1,2]. The annual incidence in Russia is 65, and the prevalence is 234.8 per 100,000 people [3]. Plaque psoriasis (i.e., psoriasis vulgaris—PV) is the most common variant of psoriasis, characterized by erythematous scaly patches or plaques that commonly occur on extensor surfaces. However, it can also affect intertriginous areas, palms, soles, and nails. The condition is often associated with a wide range of co-morbidities such as obesity, psoriatic arthritis, inflammatory bowel disease, cardiovascular, and psychosocial conditions that impair quality of life [1].

PV has a strong genetic component, as was confirmed by twin and family studies in populations of European descent. Estimates of heritability ranged from 50% to 90% [4]. Linkage analysis and the first genome-wide association study (GWAS) identified several loci as risk factors for onset PV. Most associations were found in genomic regions related with the immune system, i.e., major histocompatibility complex, signal transduction, apoptosis, cytokines, and receptors [5,6,7,8,9,10,11]. Recent data from meta-analysis revealed more than 80 loci associated with PV, which struck a multifactor nature of PV [2].

Combining multiple loci with modest effects into a global genetic risk score (GRS) might improve the identification of persons who are at risk for common complex diseases. Chen et al. were the first to develop such evaluation criteria for PV predisposition [12]. The GRS captured considerably more risk than any single-nucleotide polymorphism (SNP) considered alone. The area under curve (AUC) was chosen as the basic evaluation criteria for GRS fitness. Several subsequent GRS studies were based on the increasing number of loci associated with PV. The AUC rose to 0.8225 using 14 SNPs in the Chinese population [13]. The AUC reached 0.789 using just 5 SNPs in the Polish population [14]. For populations of Caucasian origin, the AUC achieved 0.76 using 63 SNPs [13]. A recent study created a PV prediction GRS based on 38 highly associated SNP [15]. In this study, we used GRS to differentiate patients with severe and moderate-to severe PV from the general Russian population.

The choice of PV treatment is based on the severity of the disease, which is determined according to the extent of the diseases and involvement of sensitive areas such as the face, scalp, and genital areas. The severity is roughly divided into mild, moderate, and severe, but in reality, overlap exists. Generally speaking, for mild PV treatment, various topical preparations for moderate PV phototherapy and oral systemic medications, as well as for severe PV orals, systemic medications and newer biological agents are used [16].

The understanding of PV molecular pathogenesis leads to the creation of target therapy approaches [1]. One such target drug is apremilast, which is the only selective phosphodiesterase 4 (PDE4) inhibitor approved by the U.S. Food and Drug Administration (FDA) and European Commission. Further, it is the only selective PDE4 inhibitor approved in Russia to treat moderate-to-severe and severe plaque psoriasis [17]. PDE4 inhibition, specifically hydrolyze cyclic adenosine monophosphate, leads to the cAMP intracellular level elevation [18]. Thus, the pro-inflammatory response downregulates within the T helper 1, T helper 17, and interferon pathways, all of which are pivotal in PV pathogenesis [19]. This, in turn, downregulates the production of pro-inflammatory cytokines, such as TNF-α, IL-2, IL-8, IL-12, IL-23, and IFN-γ. As such, this suppresses the PV pathway. On the other hand, PDE4 inhibition was reported to suppress the production of the IL-10 anti-inflammatory mediator and to increase the production of IL-6, which has both pro- and anti-inflammatory characteristics [20]. The side effects of apremilast are caused by PDE4 inhibition, which affects other organs (i.e., the adipose tissue) that may lead to weight loss and gastrointestinal symptoms (i.e., nausea, vomiting, and diarrhea). This most commonly occurs in the first week of therapy [17,19].

Apremilast is a safe and efficacious drug that is well tolerated. It has been shown that 33% of patients receiving apremilast achieved 75% or greater reduction from baseline versus those assigned to the placebo arm at week 16, whereas 61% patients achieved 75% at week 32 versus those assigned to the placebo. Almost the same figures were obtained during the clinical characterization of patients enrolled in the present study [3,20]. Considering the drug’s long-term application, the expense of apremilast therapy, and the differences in drug response, it is reasonable to find personalized approaches for apremilast administration.

As confirmed by many studies, genetic polymorphisms are important factors for individual variations in drug responses [21,22]. One pharmacogenomic approach is SNP association. Certain studies, however, have found different clinical outcomes [23]. The aim of the present study was to assess the influence of genomic variability on PV apremilast therapy outcomes. Since the nature of psoriasis is complicated and there is a wide spectra influence of cyclic adenosine monophosphate (cAMP) levels, we used whole genome SNP coverage analysis based on Illumina Infinium Global Screening Array-24 v2.0. To search genomic regions that contain SNPs associated with a multifactor disease, two common approaches were used: candidate SNPs and genome-wide association. In this study, we explored apremilast target therapy pharmacogenomics considering the differences between patient groups with distinct clinical outcomes for both genome-wide associations and pre-selected PV-associated SNP set.

## 2. Materials and Methods

### 2.1. Clinical Characteristics of Patients

**Figure 1:**
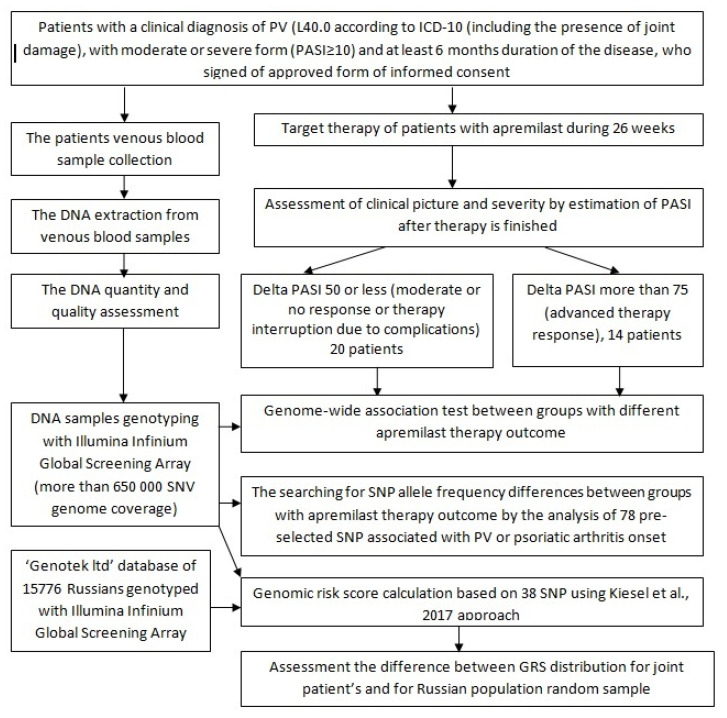
The study design. Abbreviations are: ICD-10, International Statistical Classification of Diseases and Related Health Problems according to World Health Organization; DNA, deoxyribonucleic acid.

Phenotype classification involved subjects to be directly inspected by a dermatology specialist. The recruitment of patients was based on the following inclusion criteria: individuals over the age of 18 with a clinical diagnosis of psoriasis vulgaris (including the presence of joint damage) in a moderate or severe form (PASI ≥ 10) and at least 6 months duration of the disease (Figure 1). The exclusion criteria were as follows: other forms of PV (e.g., erythrodermic, guttate, or pustular psoriasis); drug-induced PV (e.g., onset or current exacerbation of the disease due to beta-blockers, calcium channel blockers, or lithium treatment); previous use of any drugs that directly affected interleukins or their receptors within 90 days prior to the study; presence of skin diseases other than PV (such as eczema) that may interfere with clinical assessment; pregnancy or lactation (breastfeeding), where pregnancy is defined as a woman’s condition after conception and before the termination of pregnancy, confirmed by a positive human chorionic gonadotropin blood test; somatic diseases (i.e., metabolic, hematological, neurological, endocrine, infectious diseases, and diseases of the liver, kidneys, lungs, heart, or gastrointestinal tract), which significantly reduced the patient’s immunity; uncontrolled arterial hypertension; the presence of lymphoproliferative or any other malignant neoplasms [3]. The clinical assessment of PV severity was carried out via PASI (Psoriasis Area Severity Index), degree of induration, desquamation, erythema, duration of exacerbation, and the presence of complications (PA).

The PASI score was calculated as follows: four anatomical regions (head, upper limbs, trunk, and lower limbs) were assessed according to several indicators (i.e., erythema, infiltration, and desquamation). The severity of skin damage was rated on a 5-point scale: 0—absence, 1—mild, 2—moderate, 3—severe, 4—very severe. The percentage area affected by PV in a separate anatomical region was evaluated as a percentage of the total area of a given anatomical region. Afterward, it was assigned a numerical value in accordance with the degree of psoriatic lesion (from 0 to 6): (0)—0%, (1)—1–9%, (2)—10–29%, (3)—30–49%, (4)—50–69%, (5)—70–89%, (6)—90–100%. The PASI score for each body area was calculated by multiplying the sum of the severity by the area affected; the result was multiplied by the coefficient corresponding to the given anatomical region (head—0.1; trunk—0.3; upper limbs—0.2; lower limbs—0.4). Skin lesions were considered mild if PASI was <10, moderate if PASI was <20, and severe if PASI was ≥20.

### 2.2. Target Psoriasis Therapy

The monotherapy of the selective PDE4 inhibitor apremilast (Otezla^®^, Celgene International Sarl, Boudry, Switzerland) was carried out for all patients according to the Russian Federal Clinical Recommendations on PV patient treatment. The drug was prescribed in the form of tablets at a dose of 30 mg, taken orally 2 times a day (morning and evening) with an interval of about 12 h. In accordance with the manufacturer’s recommendations, during the first 5 days of therapy, the dose was titrated with a stepwise increase from 10 mg to 30 mg. A clinical assessment of PDE-4 inhibitor efficacy was carried out using delta PASI indices (in percent) at week 26 after the beginning of targeted therapy. In order to search for probable predictors of response to apremilast with high effectiveness of targeted therapy, two comparison groups were formed based on the therapy outcome: (delta PASI 75% values and higher; 14 patients) and with its insufficient effectiveness (delta PASI less than 75%; 20 patients).

### 2.3. Sample Collection

A total of 34 blood samples were collected from the ulnar vein of PV patients living in different regions of Russia. The DNA samples were extracted from venous-blood leukocytes using a QIAmp DNA Mini Kit (Qiagen, Hilden, Germany). The quality of DNA samples was assessed using agarose gel electrophoresis, and the DNA quantity was determined using Qubit 3.0. All samples obtained were confirmed to be suitable for genotyping.

### 2.4. Genotyping

Samples were prepared using Illumina Infinium Global Screening Array-24 v2.0 (Illumina, San Diego, CA, USA). Array scanning on the HiScan (Illumina) platform were conducted according to the Infinium HTS Assay Guide. Genotyping was created using Illumina Auto Convert Software v2.0.1 (Illumina, San Diego, CA, USA). An Autosomal Call Rate Threshold equal to 0.01 was selected. Genotyping quality was assessed using call rate, and it appeared to be higher than 0.99 in all analyzed samples. Allele designations followed the Genome Reference Consortium Human Build 38 (GRCh38) (https://www.ncbi.nlm.nih.gov/assembly/GCF_000001405.38).

### 2.5. Data Analyses

The data obtained were inputwith BEAGLE 5.0 software, which had a Haplotype Reference Consortium reference panel and was filtered with DR2 > 0.7. An association test was conducted using PLINK v1.9 (classical filters were applied: geno 0.05, maximum percentage of missed values of polymorphisms; mind 0.1, maximum percentage of missed sample values; hwe 1e-5, exclusion of the polymorphisms (*p*-value threshold was 1e*^−5^) deviated significantly from Hardy–Weinberg equilibrium; mac 5, exclusion of SNPs by MAF (minimal number of minor alleles in a cohort is five)). The list of SNPs with statistically significant distinctions between patient groups with moderate or effective therapy outcomes had a coincidence probability less than 10^−5^ and a represented in Supplementary Table S1. The assessment of allele frequency differences at the pre-selected SNP sets between groups was conducted using the χ2 probability test. Therein, we found that the consuming significance probability level was 0.05 (Table 1). The polygenic risk score was 38 SNP and was calculated according to Kisiel et al. [15]. AUC values were calculated with the R-package “pROC” software. The random Russian population sample of 15,776 individuals was consulted with Genotek Ltd. and accordingly genotyped to be used as the control group [24].

## 3. Results and Discussion

It should be noted that the effectiveness of therapy in this study was slightly lower than in international clinical trials and real-world studies [61,62,63,64,65]. So, 44% of patients did not reach PASI 50 by 14 weeks, and by 26 weeks—47%. This is probably due to the fact that more than half (53%) of the patients included in the study had severe psoriasis, and also had a burdened history in the form of the duration of the disease and the ineffectiveness of previous therapy.

**Figure 2:**
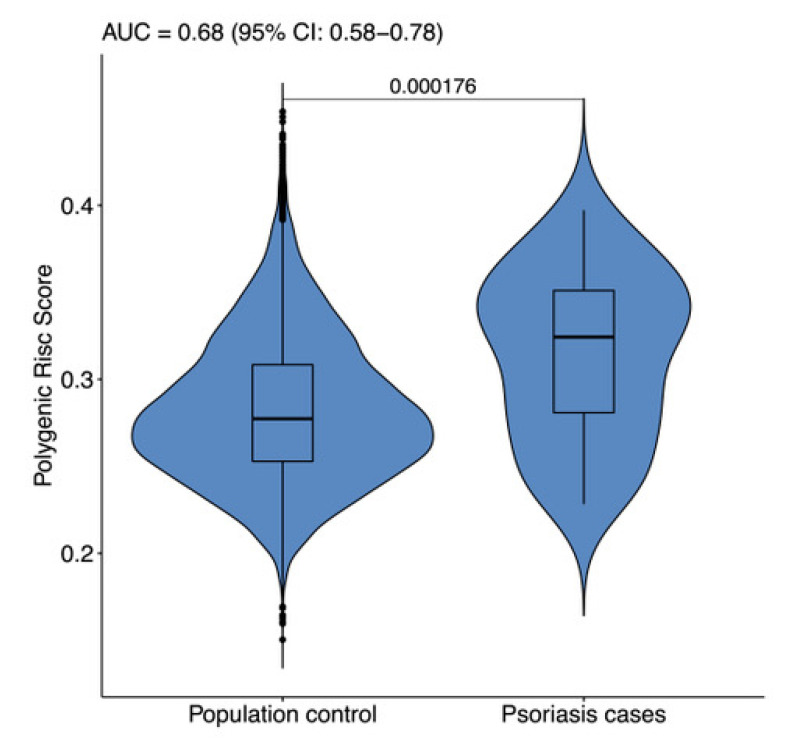
38 SNP genetic risk score (GRS) [15] distribution violin plots for the general Russian population sample (Genotek clients; sample size = 15,776) vs. moderate-to-severe and severe psoriasis patients. The area under curve (AUC) value for the GRS was 0.6821 (95% CI: 0.5816–0.7837). Mann–Whitney–Wilcoxon *p*-value indicated above the line.

Before the pharmacogenomic analysis, we checked whether the cohort possessed genome peculiarities that differed from the general Russian population. To evaluate the difference, GRS with 38 SNP were calculated both in the patient cohort (34 patients with moderate-to-severe and severe psoriasis) and in the population control group according to Kiesel et al. [15]. Figure 2 indicates violine plots obtained for the following groups: AUC value assessment for the differentiation was 0.6821 (95% CI: 0.5816–0.7837) and significantly differed from AUC = 0.5 for the non-informative model (Mann–Whitney–Wilcoxon *p*-value was 0.000175). This difference level suggested the possibility of using GRS to determine PV predisposition. In the original Kiesel et al. study [15], the AUC value for wGRS reached 0.782. Therefore, the lower AUC in this study can be explained by our low cohort sample size or by the inclusion of individuals with early stage PV. The usefulness of GRS for assessing an individual’s PV predisposition does not mean that the same GRS is suitable for finding differences in patient cohorts with different clinical outcome of apremilast treatment. Calculating GRS in these patient groups revealed no distribution differences, thus uncovering nonsignificant influence of the genomic regions associated with PV predisposition on target treatment outcomes (Mann–Whitney–Wilcoxon *p*-value was 0.8766. AUC was 0.5179).

The association test—which based all genetic data obtained with the Illumina Infinium Global Screening Array from patient cohorts with different clinical outcomes of apremilast treatment—revealed genome regions associated with therapy effectiveness. The restricted list of associations, limited by the 10^−6^ *p*-value, was provided in Table S1. In detail, 72 SNPs, situated on four chromosomes, were associated with differences in PV apremilast therapy outcomes. There were11 SNPs at chromosome 2, covering 37 kbp region; 54 SNPs at chromosome 4 covered 44 kbp region; 6 SNPs at chromosome 5 covered6.4 kbp region and the only SNP at X-chromosome. These regions situated at the genome loci with an unrecognized function were not situated at or nearby certain genes, yet SNP rs35084576 at X-chromosome was in the *ARSF* (aryl sulfatase F gene). At first glance, SNP flanking regions show no remarkable peculiarities; however, SNP rs371208912 on chromosome 5 possesses minimal *p*-value, as it flanks the region with mutations. Therefore, extended deletions can occur (Live Ref SNPs, dbSNPb154v2, NCBI, 2020) (https://www.ncbi.nlm.nih.gov/snp/docs/RefSNP_about/). This region might be considered as a mutation hot point; however, further studies are required to resolve this issue. According to the genome-wide association analysis, there were a few genome regions highly associated with different apremilast therapy clinical outcomes. Considering the low sample size of the patient cohort, it was hard to form a haplotype analysis within the regions due to the high level of possible misinterpretation. It was also difficult to speculate about the less significant genome regions because of the huge amount of associations found. However, group differences, confirmed with a probability of 10^−6^, led us to conclude that our data can be used for a pilot model to predict apremilast therapy outcomes.

To explore the genomic regions associated with apremilast clinical outcomes, 78 SNPs strongly associated with PV or psoriatic arthritis were selected from the available sources [26,27,28,29,30,31,32,33,34,35,36,37,38,39,40,41,42,43,44,45,46,47,48,49,50,51,52,53,54,55,56,57,58,59,60]. The SNP set included genomic regions from cytokine genes (*IL1A, IL1B, IL2, IL6, IL10, IL10B, IL4 (IL13), IL17, IL17F, IL17RA, IL22, IL23R, TNF, TNFAIP3*), enzymes (Tyrosine kinase 2 *(TYK2)*), phoshodiesterases *(PDE3D* and *PDE4D),* and signal transduction activation factors (*STAT(1–4))*, as well some other signaling molecules (*NOS2, NFkB, REL, ZNF313, IFNγ,* and detoxification enzyme *CYP3A5* (https://www.pharmgkb.org/vip/PA166170041 (Very Important Pharmacogene: CYP3A5)) (Table 1). The strongest associations found in the genome-wide association showed no similarity with regions established for pre-selected SNP. The associations in the allele frequency distribution with apremilast target therapy outcomes were found for SNPs situated at *IL1β* (rs1143633, *p* = 0.05, χ2 = 3.82; OR = 2.69), *IL4 (IL13*) (rs20541, *p* = 0.04, χ2 = 4.15; OR = 3.00), *IL23R* (rs2201841, *p* = 0.03, χ2 = 4.51, OR = 4.51), and *TNFα* (rs1800629, *p* = 0.03, χ2 = 4.83; OR = 8.48) genes. In all cases, the better outcome was revealed in the patient group with increased minor allele frequency. The analysis of the allele frequency of the SNP set showed a coincidence with the European population cohort data from 1000 Genomes. This served as an example of population genomic variability, which decreased psoriasis SNPs prediction value in Russians. Thus, the SNP allele frequencies associated best with apremilast clinical outcomes were deviated from allele distribution of healthy populations, whereas the patient group with lowered or no apremilast efficiency allele frequencies were equal to or approaching those.

Cytokines IL-1*β* and TNFα are pro-inflammatory cytokines produced by activated macrophages [66]. *IL23R* encodes a subunit of the receptor required for IL23A signaling. This protein associates constitutively with JAK2 and binds to transcription activator STAT3, which is one of the key signaling molecules for PV [67]. *IL13* encodes a cytokine produced by activated Th2 that is involved in the maturation and differentiation of B cells. *IL13* downregulates macrophage activity and inhibits proinflammatory cytokine and chemokine production. Along with the psoriasis vulgaris association, *IL1β* SNP rs1143633 is associated to eczema, hay fever, and asthma [68]. *IL13* SNP rs20541has also been found to be associated with allergies, hay fever, asthma, and eczema [69]. *IL23R* SNP rs2201841 is associated with Crohn’s disease [70]. *TNFα*(-308) SNP rs1800629 is associated with several immune diseases, i.e., asthma, Crohn’s disease, system lupus erithematosus, Mediterranean spotted fever, multiple sclerosis, nasal polypes, lymphoma, leprosy, chronic obstructive pulmonary disease, and heart disease (https://www.snpedia.com/index.php/Rs1800629). Thus, the SNPs associated with apremilast therapy outcomes were also associated with immune polygenic diseases [71]. Recent studies on immunometabolism (i.e., the specific function of metabolites in immune system regulation via Krebs cycle rewiring [72]) uncovered the interconnection of intercellular metabolites (e.g., succinate, lactic acid, itakonate) levels on IL-1*β* and TNFα production during inflammation, as well the influence of α-ketoglutarate on the macrophage polarization process by changing *IL4(IL13)* dependent gene transcription [73]. The authors considered anti-inflammation effects for several drugs used in PV and RA treatment, such as dimethyl fumarate, metformin, and methotrexate. Since apremilast is an anti-inflammation drug that downregulates pro-inflammatory cytokine production after cAMP levels increase, it may also influence inflammation by changing metabolite concentration. The clinical application of apremilast results in better PA outcomes than PV outcomes. The succinate level was elevated in synovial fluid from RA patients [72] and it may be hypothesized that apremilast affects immunometabolism. SNP associations with clinical outcomes revealed indirect influences of the drug on associated cytokine levels due to cell metabolism modification. According to Zaslona and O’Neill, “We are still in the pioneering phase of gathering information about the functions of specific metabolites in immunoregulation” [73]. This offers hope for progress and future development regarding apremilast’s influence on PV immunopathogenesis and therapy outcomes. An alternative way to solve the issue is genomic transcription study. cAMP signaling associated with G-protein coupled receptors (GPCR-cAMP-PKA pathway) [74], including CREBs (cAMP-response element binding protein) regulation of gene transcription under apremilast action [75].

## 4. Conclusions

Although understanding genetic backgrounds isjust a part of psoriasis pathogenesis [2], the associations found in this study will pave the way for the future creation of a model used in apremilast therapy outcome prediction. Moreover, the validation of GRS created for 38 SNPs was found to be applicable for PV susceptibility in Russians. The only obstacle to creating this model was the low patient cohort sample size used in this study. The sample size should be expanded in the future.

## Figures and Tables

**Table 1:** The data obtained for 78 pre-selectedsingle-nucleotide polymorphisms(SNPs) [25,26,27,28,29,30,31,32,33,34,35,36,37,38,39,40,41,42,43,44,45,46,47,48,49,50,51,52,53,54,55,56,57,58,59,60] in patient groups with advanced and lowered target apremilast therapy clinical outcomes. SNP with statistically significant differences are labeled with a star *. The population was from European origin and were healthy. The“1000 Genomes” project is shown for reference.

Gene or Nearby Genomic Region	SNP Reference Sequence	Most Frequent Allele Variant	Minimal Frequency Allele Variants	Allele Frequency in Efficient Target Therapy Response Group	Allele Frequency in Lowered Target Therapy Response Group	Allele Frequency in the Population of European Origin	References
*IL1A, IL1B*	rs12469600	T	C	0.79	0.70	0.752	[25]
*IL1B*	rs1143633 *	C	T	0.46	0.70	0.666	[29]
*IL1B*	rs11676014	G	A	0.68	0.60	0.551	[26]
*IL1B* 2KB upstream	rs16944	G	A	0.75	0.55	0.65	[28]
*IL1B* 500B downstream	rs2853550	G	A	0.96	0.87	0.954	[28]
*IL2* 2KB upstream	rs2069762	A	C	0.75	0.62	0.708	[31]
*IL2*	rs2069778	G	A	0.64	0.77	0.849	[31]
*IL-6*	rs1800795	C	G	0.46	0.43	0.444	[32]
*IL10* 2KB upstream	rs1800871	G	A	0.79	0.62	0.76	[34]
*IL10* 2KB upstream	rs1800872	G	T	0.79	0.62	0.76	[34]
*IL10* 2KB upstream	rs1800896	T	C	0.50	0.62	0.547	[33]
*IL10RA*	rs947889	T	C	0.46	0.62	0.54	Live Ref SNPs
noncoding (*IL-12B*)	rs2082412	G	A	0.89	0.85	0.774	[35]
*IL13, IL4*	rs1800925	C	T	0.71	0.75	0.822	[36]
*IL13, IL4*	rs20541 *	G	A	0.57	0.80	0.793	[36]
*IL4* 2KB upstream	rs2243250	C	T	0.57	0.75	0.832	[59]
*IL13* 3′UTR	rs848	C	A	0.57	0.77	0.787	[36]
*IL17A*	rs10484879	G	T	0.93	0.82	0.75	[37]
*IL17A* 2KB upstream	rs2275913	G	A	0.75	0.55	0.62	[37]
*IL-17F*	rs763780	T	(C)	0.89	1	0.942	[37]
*IL17RA:* 2KB upstream	rs4819554	A	G	0.79	0.90	0.792	[37]
*IL22*	rs12307915	T	C	0.86	0.77	0.808	[58]
*IL22* 2kb upstream	rs2227473	C	T	0.86	0.77	0.81	[58]
*IL22* 2kb upstream	rs2227483	A	T	0.46	0.60	0.547	[58]
*IL23R* 500B downstream	rs9988642	T	C	0.96	0.95	0.928	[38]
*IL23R*	rs12564022	C	T	0.61	0.72	0.702	[60]
*IL-23R*	rs11209026	G	A	0.96	0.95	0.938	[42]
*IL23R*	rs2201841 *	A	G (T)	0.5	0.75	0.7	[35]
*IL23R*	rs2295359	G	A	0.64	0.55	0.686	[60]
*IL23R*	rs11209032	G	A	0.57	0.65	0.666	[42]
*IL28RA*	rs4649203	A	G	0.82	0.77	0.725	[48]
*TYK2*	rs12720356	A	C(G)	0.96	0.97	0.908	[43]
*TYK2*	rs280519	A	G(C)	0.50	0.55	0.505	[43]
*TYK2*	rs2304256	C	A	0.79	0.80	0.738	[43]
*TYK2*	rs34536443	G	C	1	1	0.971	[43]
*PDE3A-SLC01C1*	rs3794271	A	G	0.54	0.60	0.635	[44,45]
*PDE4D (PART1)*	rs152312	G	(A, T)	0.96	0.92	0.9	[46]
*PDE4D*	rs2910829	A	G	0.71	0.52	0.56	[46]
*TNFa*	rs1799724	C	T	0.79	0.82	0.906	[37]
*TNFa*	rs1799964	T	C	0.82	0.72	0.79	[47]
*TNFa*	rs1800629 *	G	A	0.82	0.97	0.866	[47]
*TNF* 2KB upstream	rs361525	G	A	0.93	0.92	0.936	[47]
*TNFAIP3*	rs610604	T	G	0.64	0.67	0.662	[37]
*STAT1*	rs2083482	T	C	0.71	0.62	0.496	[57]
*STAT2*	rs2020854	T	C	0.93	0.90	0.931	[53]
*STAT2*	rs2066807	C	G	0.93	0.90	0.933	[8]
*STAT2*	rs2066808	A	G	0.93	0.90	0.93	[58]
*STAT3*	rs2293152	C	G	0.50	0.60	0.596	[27]
*STAT3*	rs8074524	C	T	0.86	0.77	0.8	Live Ref SNPs
*STAT3*	rs744166	A	G	0.68	0.55	0.585	[56]
*STAT4*	rs7574865	G	T	0.71	0.85	0.77	[52]
*STAT4*	rs10181656	C	G	0.71	0.85	0.766	[52]
*NFkB1*	rs28362491	INS	DEL	0.54	0.60	0.595	[54]
*NF-kB1A*	rs2145623	G	C	0.82	0.70	0.724	[30]
*NF-kB1A*	rs8016947	T	G	0.50	0.47	0.471	[55]
*NFkB1A*	rs12586317	T	C	0.61	0.65	0.737	[41]
*REL*	rs62149416	T	C	0.61	0.80	0.636	[53]
*REL*	rs702873	C	T	0.50	0.70	0.553	[52]
*RNF114*	rs495337	G	A	0.61	0.62	0.602	[41]
*ZNF313*	rs2235617	C	G	0.61	0.65	0.602	[39]
*REV3L*	rs240993	C	A	0.71	0.55	0.712	[30]
*IFNG*	rs2430561	A	T	0.54	0.40	0.462	[51]
*CAST, ERAP1*	rs27524	G	A	0.54	0.65	0.634	[48]
*HLA-C*	rs4406273	G	A	0.79	0.72	0.902	[50]
*NOS2*	rs4795067	A	G	0.50	0.57	0.641	[41]
*CYP3A5*	rs776746	C	T	1	0.92	0.943	PharmGKB
*TSC1*	rs1076160	C	T	0.71	0.52	0.489	[55]
*FBXL19*	rs10782001	A	G	0.54	0.62	0.641	[41]
*KCNH7*	rs17716942	T	C	0.96	0.90	0.839	[39]
*ANXA6*	rs17728338	G	A	0.96	0.87	0.93	[35]
*HCP5*	rs2395029	T	G	0.93	0.92	0.956	[40]
*COG6*	rs7993214	C	T(G)	0.57	0.62	0.657	[40]
*SDC4*	rs1008953	C	T	0.86	0.72	0.775	[41]
*RPS26*	rs12580100	A	G	0.93	0.85	0.867	[41]
NONE	rs1975974	A	G	0.82	0.77	0.763	[41]
*LCE3D*	rs4112788	G	A	0.68	0.62	0.644	[8]
*LOC105376976*	rs6809854	A	G	0.64	0.77	0.802	[41]
*LOC107984144*	rs10484554	C	T	0.68	0.60	0.857	[40]

## Data Availability

The genotypes dataset obtained is available on inquire from corresponding author.

## Supplementary Materials

The following are available online at https://www.mdpi.com/2075-4426/11/1/20/s1. Table S1: List of top SNPs that resulted from the association test of genome-wide pharmacogenomic studies on apremilast between patients groups with distant therapy outcomes.

## Abbreviations

AUC	Area under the curve
cAMP	Cyclic adenosine monophosphate
GRC	Global genetic risk score
GWAS	Genome-wide association studies
PASI	Psoriasis area severity index
PDE4	phosphodiesterase 4
PA	Psoriasis arthritis
PV	Psoriasis vulgaris
RA	Rheumatoid arthritis
STAT	signal transduction and activator of transcription

## References

[1] Armstrong A.W., Read C. (2020). Pathophysiology, Clinical Presentation and Treatment of Psoriasis. A Review. JAMA.

[2] Ogawa K., Okada Y. (2020). The current landscape of psoriasis genetics in 2020. J. Dermatol. Sci..

[3] Kubanov A.A., Artamonova O.G., Karamova A.E., Vasileva E.L., Deryabin D.G. (2020). Skin Lesions Cytokines in Moderate and Severe Psoriasis as Predictors for the Type 4 Phosphodiesterase inhibitor (Apremilast) Therapy Effectiveness. Ann. Russ. Acad. Med. Sci..

[4] Stuart P.E., Nair R.P., Tsoi L.C., Tejasvi T., Das S., Kang H.M., Ellinghaus E., Chandran V., Callis-Duffin K., Ike R. (2015). Genome-wide Association Analysis of Psoriatic Arthritis and Cutaneous Psoriasis Reveals Differences in Their Genetic Architecture. Am. J. Hum. Genet..

[5] Woodrow J.C., Ilchysyn A. (1985). HLA antigens in psoriasis and psoriatic arthritis. J. Med. Genet..

[6] Elder J.T., Nair R.P., Guo S.-W., Henseler T., Christophers E., Voorhees J.J. (1994). The Genetics of Psoriasis. Arch. Derm..

[7] Elder J.T., Nair R.P., Henseler T., Jenisch S., Stuart P., Chia N., Christophers E., Voorhees J.J. (2001). The Genetics of Psoriasis 2001: The Odyssey Continues. Arch. Derm..

[8] Nair R.P., Duffin K.C., Helms C., Ding J., Stuart P.E., Goldgar D., Gudjonsson J.E., Li Y., Tejasvi T., Feng B.-J. (2009). Genome-wide scan reveals association of psoriasis with IL-23 and NF-κB pathways. Nat. Genet..

[9] Nair R.P., Ruether A., Stuart P.E., Jenisch S., Tejasvi T., Hiremagalore R., Schreiber S., Kabelitz D., Lim H.W., Voorhees J.J. (2008). Polymorphisms of the IL12B and IL23R genes are associated with psoriasis. J. Invest. Derm..

[10] Julia A., Tortosa R., Hernanz J.M., Canete J.D., Fonseca E., Ferrandiz C., Unamuno P., Puig L., Fernandez-Sueiro J.L., Sanmarti R. (2012). Risk variants for psoriasis vulgaris in a large case-control collection and association with clinical subphenotypes. Hum. Mol. Genet..

[11] Tsoi L.C., Stuart P.E., Tian C., Gudjonsson J.E., Das S., Zawistowski M., Ellinghaus E., Barker J.N., Chandran V., Dand N. (2017). Large scale meta-analysis characterizes genetic architecture for common psoriasis associated variants. Nat. Commun..

[12] Chen H., Poon A., Yeung C., Helms C., Pons J., Bowcock A.M., Kwok P.Y., Liao W. (2011). A genetic risk score combining ten psoriasis risk loci improves disease prediction. PLoS ONE.

[13] Yin X., Cheng H., Lin Y., Wineinger N.E., Zhou F., Sheng Y., Yang C., Li P., Li F., Shen C. (2015). A Weighted Polygenic Risk Score Using 14 Known Susceptibility Variants to Estimate Risk and Age Onset of Psoriasis in Han Chinese. PLoS ONE.

[14] Stawczyk-Macieja M., Rębała K., Szczerkowska-Dobosz A., Wysocka J., Cybulska L., Kapińska E., Haraś A., Miniszewska P., Nowicki R. (2016). Evaluation of psoriasis genetic risk based on five susceptibility markers in a population from northern Poland. PLoS ONE.

[15] Kisiel B., Kisiel K., Szymański K., Mackiewicz W., Biało-Wójcicka E., Uczniak S., Fogtman A., Iwanicka-Nowicka R., Koblowska M., Kossowska H. (2017). The association between 38 previously reported polymorphisms and psoriasis in a Polish population: High predicative accuracy of a genetic risk score combining 16 loci. PLoS ONE.

[16] Shavit E., Shear N.H. (2020). An update on the safety of apremilast for the treatment of plaque psoriasis. Expert Opin. Drug Saf..

[17] Kubanov A.A., Karamova A.E., Artamonova O.G. (2018). New opportunities in the treatment of psoriasis and psoriatic arthritis. Rheumatol. Sci. Pract..

[18] Schett G., Sloan V.S., Stevens R.M., Schafer P. (2010). Apremilast: A novel PDE4 inhibitor in the treatment of autoimmune and inflammatory diseases. Adv. Musculoskelet Dis..

[19] Simard M., Morin S., Rioux G., Séguin R., Loing E., Pouliot R.A. (2020). Tissue-Engineered Human Psoriatic Skin Model to Investigate the Implication of cAMP in Psoriasis: Differential Impacts of Cholera Toxin and Isoproterenol on cAMP Levels of the Epidermis. Int. J. Mol. Sci..

[20] Kubanov A.A., Solomka V.S., Karamova A.E., Verbenko D.A., Vasilieva E.L., Artamonova O.G. (2020). The effect of apremilast therapy on skin cytokine levels in patients with psoriasis. Russ. Open Med. J..

[21] Giacomini K.M., Yee S.W., Mushiroda T., Weinshilboum R.M., Ratain M.J., Kubo M. (2017). Genome-wide association studies of drug response and toxicity: An opportunity for genome medicine, *Nat*. Rev. Drug Discov..

[22] Nelson M.R., Johnson T., Warren L., Hughes A.R., Chissoe S.L., Xu C.F., Waterworth D.M. (2016). The genetics of drug efficacy: Opportunities and challenges. Nat. Rev. Genet..

[23] Ovejero-Benito M.C., Prieto-Pérez R., Llamas-Velasco M., Muñoz-Aceituno E., Reolid A., Saiz-Rodríguez M., Belmonte C., Román M., Ochoa D., Talegón M. (2018). Polymorphisms associated with adalimumab and infliximab response in moderate-to-severe plaque psoriasis. Pharmacogenomics.

[24] Kozina A.A., Okuneva E.G., Baryshnikova N.V., Kondakova O.B., Nikolaeva E.A., Fedoniuk I.D., Mikhailova S.V., Krasnenko A.Y., Stetsenko I.F., Plotnikov N.A. (2020). Neuronal ceroid lipofuscinosis in the Russian population: Two novel mutations and the prevalence of heterozygous carriers. Mol. Genet. Genom. Med..

[25] Buniello A., MacArthur J.A.L., Cerezo M., Harris L.W., Hayhurst J., Malangone C., McMahon A., Morales J., Mountjoy E., Sollis E. (2019). The NHGRI-EBI GWAS Catalog of published genome-wide association studies, targeted arrays and summary statistics 2019. Nucleic Acids Res..

[26] Bidwell J., Keen L., Gallagher G., Kimberly R., Huizinga T., McDermott M.F., Oksenberg J., McNicholl J., Pociot F., Hardt C. (1999). Cytokine gene polymorphism in human disease: On-line databases. Genes Immun..

[27] Zhou J., Li Y., Sun D. (2017). Associations between STAT Gene Polymorphisms and Psoriasis in Northeastern China. Dermatology.

[28] Sasayama D., Hori H., Iijima Y., Teraishi T., Hattori K., Ota M., Fujii T., Higuchi T., Amano N., Kunugi H. (2011). Modulation of cortisol responses to the DEX/CRH test by polymorphisms of the interleukin-1beta gene in healthy adults. Behav. Brain Funct..

[29] Hebert H.L., Bowes J., Smith R.L., McHugh N.J., Barker J., Griffiths C., Barton A., Warren R. (2014). Polymorphisms in IL-1B distinguish between psoriasis of early and late onset. J. Investig. Dermatol..

[30] Prieto-Pérez R., Cabaleiro T., Daudén E., Ochoa D., Roman M., Abad-Santos F. (2013). Genetics of psoriasis and pharmacogenetics of biological drugs. Autoimmune Dis..

[31] Bouzid D., Fourati H., Amouri A., Marques I., Abida O., Tahri N., Penha-Gonçalves C., Masmoudi M. (2014). Autoimmune diseases association study with the KIAA1109-IL2-IL21 region in a Tunisian population. Mol. Biol. Rep..

[32] Białecka M., Ostasz R., Kurzawski M., Klimowicz A., Fabiańczyk H., Bojko P., Dziedziejko V., Safranow K., Droździk M. (2015). IL6 -174G>C polymorphism is associated with an increased risk of psoriasis but not response to treatment. Exp Derm..

[33] Craven N., Jackson C., Kirby B., Perrey C., Pravica V., Hutchinson I., Griffiths C. (2001). Cytokine gene polymorphisms in psoriasis. Br. J. Dermatol..

[34] Lee Y.H., Choi S.J., Ji J.D., Song G.G. (2012). Associations between interleukin-10 polymorphisms and susceptibility to psoriasis: A meta-analysis. Inflamm. Res..

[35] Elder J.T. (2009). Genome-wide association scan yields new insights into the immunopathogenesis of psoriasis. Genes Immun..

[36] Chang M., Li Y., Yan C., Callis-Duffin K.P., Matsunami N., Garcia V.E., Cargill M., Civello D., Bui N., Catanese J.J. (2008). Variants in the 5q31 cytokine gene cluster are associated with psoriasis. Genes Immun..

[37] Murdaca G., Negrini S., Magnani O., Penza E., Pellecchio M., Puppo F. (2017). Impact of pharmacogenomics upon the therapeutic response to etanercept in psoriasis and psoriatic arthritis. Expert Opin. Drug Saf..

[38] Tsoi L.C., Spain S.L., Knight J., Ellinghaus E., E Stuart P., Capon F., Ding J., Pullinger C.R., Tejasvi T., Gudjonsson J.E. (2012). Identification of 15 new psoriasis susceptibility loci highlights the role of innate immunity. Nat. Genet..

[39] Strange A., Capon F., Spencer C.C.A., Knight J., Weale M.E., Allen M.H., Barton A., Band G., Bellenguez C., Bergboer J.G.M. (2010). Genetic Analysis of Psoriasis Consortium & the Wellcome Trust Case Control Consortium; A genome-wide association study identifies new psoriasis susceptibility loci and an interaction between HLA-C and ERAP1. Nat. Genet..

[40] Liu Y., Helms C., Liao W., Zaba L.C., Duan S., Gardner J., Wise C., Miner A., MJ Malloy M.J., Pullinger C.R. (2008). A genome-wide association study of psoriasis and psoriatic arthritis identifies new disease loci. PLoS Genet..

[41] Stuart P.E., Nair R.P., Ellinghaus E., Ding J., Tejasvi T., Gudjonsson J.E., Li Y., Weidinger S., Eberlein B., Gieger C. (2010). Genome-wide association analysis identifies three psoriasis susceptibility loci. Nat. Genet..

[42] Zhu K., Zhu C.-Y., Shi G., Fan Y. (2012). Association of IL23R polymorphisms with psoriasis and psoriatic arthritis: A meta-analysis. Inflamm. Res..

[43] Tao J.-H., Zou Y.-F., Feng X.-L., Li J., Wang F., Pan F.-M., Ye D.-Q. (2011). Meta-analysis of TYK2 gene polymorphisms association with susceptibility to autoimmune and inflammatory diseases. Mol. Biol. Rep..

[44] Julia A., Rodriguez-Moreno J., Fernandez-Sueiro J.L., Gratacos J., Queiro R., Montilla C., Torre-Alonso J.C., Pérez-Venegas J.J., Manrique-Arija S., Muñoz-Fernández S. (2014). PDE3A-SLCO1C1 locus is associated with response to anti-tumor necrosis factor therapy in psoriatic arthritis. Pharmacogenomics.

[45] Osmola-Mańkowska A., Teresiak-Mikołajczak E., Skrzypczak-Zielińska M., Adamski Z. (2018). Genetic polymorphism in psoriasis and its meaning for the treatment efficacy in the future. Postepy Derm. Alergol..

[46] Newcombe P.J., Verzilli C., Casas J.P., Hingorani A.D., Smeeth L., Whittaker J.C. (2009). Multilocus Bayesian meta-analysis of gene-disease associations. Am. J. Hum. Genet..

[47] Cabaleiro T., Román M., Gallo E., Ochoa D., Tudelilla F., Talegón M., Prieto-Pérez R., García-Díez A., Daudén E., Abad-Santos F. (2013). Association between psoriasis and polymorphisms in the TNF, IL12B, and IL23R genes in Spanish patients. Eur. J. Derm..

[48] Yang Q., Liu H., Qu L., Fu X., Yu Y., Yu G., Tian H., Yu Y., Sun D., Peng J. (2013). Investigation of 20 non-HLA (human leucocyte antigen) psoriasis susceptibility loci in Chinese patients with psoriatic arthritis and psoriasis vulgaris. Br. J. Dermatol..

[49] Bowes J., Orozco G., Flynn E., Ho P., Brier R., Marzo-Ortega H., Coates L., McManus R., Ryan A.W., Kane D. (2011). Confirmation of TNIP1 and IL23A as susceptibility loci for psoriatic arthritis. Ann. Rheum. Dis..

[50] Stuart P.E., Tejasvi T., Shaiq P.A., Kullavanijaya P., Qamar R., Raja G.K., Li Y., Voorhees J.J., Abecasis G.R., Elder J.T. (2015). A Single SNP Surrogate for Genotyping HLA-C*06:02 in Diverse Populations. J. Investig. Dermatol..

[51] Popadic S., Savic E., Markovic M., Ramic Z., Medenica L., Pravica V., Spuran Z., Trajkovic V., Popadic D. (2015). TNF, IL12B, and IFNG Gene Polymorphisms in Serbian Patients with Psoriasis. Ann. Derm..

[52] Bowes J., Ho P., Flynn E., Ali F., Marzo-Ortega H., Coates L.C., Warren R.B., McManus R., Ryan A.W., Kane D. (2012). Comprehensive assessment of rheumatoid arthritis susceptibility loci in a large psoriatic arthritis cohort. Ann. Rheum. Dis..

[53] Bowes J., Budu-Aggrey A., Huffmeier U., Uebe S., Steel K., Hebert H.L., Wallace C., Massey J., Bruce I.N., Bluett J. (2015). Dense genotyping of immune-related susceptibility loci reveals new insights into the genetics of psoriatic arthritis. Nat. Commun..

[54] Caldarola G., Sgambato A., Fanali C., Moretta G., Farina M., Lucchetti D., Peris K., De Simone C.D. (2016). HLA-Cw6 allele, NFkB1 and NFkBIA polymorphisms play no role in predicting response to etanercept in psoriatic patients. Pharm. Genom..

[55] Li Y., Cheng H., Zuo X., Sheng Y.-J., Zhou F., Tang X.-F., Tang H.-Y., Gao J.-P., Zhang Z., He S.-M. (2013). Association analyses identifying two common susceptibility loci shared by psoriasis and systemic lupus erythematosus in the Chinese Han population. J. Med. Genet..

[56] Kim S.Y., Hur M.S., Choi B.G., Kim M.J., Lee Y.W., Choe Y.B., Ahn K.J. (2017). A preliminary study of new single polymorphisms in the T helper type 17 pathway for psoriasis in the Korean population. Clin. Exp. Immunol..

[57] Swindell W.R., Sarkar M.K., Stuart P.E., Voorhees J.J., Elder J.T., Johnston A., Gudjonsson J.E. (2015). Psoriasis drug development and GWAS interpretation through in silico analysis of transcription factor binding sites. Clin. Transl. Med..

[58] Nikamo P., Lysell J., Ståhle M. (2015). Association with Genetic Variants in the IL-23 and NF-κB Pathways Discriminates between Mild and Severe Psoriasis Skin Disease. J. Investig. Dermatol..

[59] Smolnikova M.V., Freidin M.B., Barilo A.A., Smirnova S.V. (2019). Analysis of association between cytokine gene polymorphisms and psoriatic disease in Russians of East Siberia. Meta Gene.

[60] Yin X., Low H.Q., Wang L., Li Y., Ellinghaus C.E.E., Han J., Estivill X., Sun L., Zuo X., Shen C. (2015). Genome-wide meta-analysis identifies multiple novel associations and ethnic heterogeneity of psoriasis susceptibility. Nat. Commun..

[61] Papp K., Reich K., Leonardi C.L., Kircik L., Chimenti S., Langley R.G., Korman N.J. (2015). Apremilast, an oral phosphodiesterase 4 (PDE4) inhibitor, in patients with moderate to severe plaque psoriasis: Results of a phase III, randomized, controlled trial (Efficacy and Safety Trial Evaluating the Effects of Apremilast in Psoriasis [ESTEEM] 1). J. Am. Acad. Dermatol..

[62] Paul C., Cather J., Gooderham M., Poulin Y., Mrowietz U., Ferrandiz C., Day R.M. (2015). Efficacy and safety of apremilast, an oral phosphodiesterase 4 inhibitor, in patients with moderate-to-severe plaque psoriasis over 52 weeks: A phase III, randomized controlled trial (ESTEEM 2). Br. J. Dermatol..

[63] Vujic I., Herman R., Sanlorenzo M., Posch C., Monshi B., Rappersberger K., Richter L. (2018). Apremilast in psoriasis—A prospective real-world study. J. Eur. Acad. Dermatol. Venereol..

[64] Augustin M., Kleyn C.E., Conrad C., Sator P.G., Ståhle M., Eyerich K., Cordey M. Characteristics and Outcomes of Patients Treated With Apremilast in the Real World: Results From the APPRECIATE Study. J. Eur. Acad. Dermatol. Venereol..

[65] De A., Das S., Dhoot D., Sarda A. (2020). Real-World Insight on Apremilast Therapy in Patients with Plaque Psoriasis: Indian Experience. Indian J. Derm..

[66] Calautti E., Avalle L., Poli V. (2018). Psoriasis: A STAT3-Centric View. Int. J. Mol. Sci..

[67] Reich K., Mossner R., Konig I.R., Westphal G., Ziegler A., Neumann C. (2002). Promoter polymorphisms of the genes encoding tumor necrosis factor-α and interleukin-1β are associated with different subtypes of psoriasis characterized by early and late disease onset. J. Investig. Dermatol..

[68] Johansson Å., Rask-Andersen M., Karlsson T., Ek W.E. (2019). Genome-wide association analysis of 350,000 Caucasians from the UK Biobank identifies novel loci for asthma, hay fever and eczema. Hum. Mol. Genet..

[69] Black S., Teixeira A.S., Loh A.X.W., Vinall L., Holloway J.W., Hardy R., Swallow D.M. (2009). Contribution of functional variation in the IL13 gene to allergy, hay fever and asthma in the NSHD longitudinal 1946 birth cohort. Allergy.

[70] Safrany E., Szabo M., Szell M., Kemeny L., Sumegi K., Melegh B.I., Magyari L., Matyas P., Figler M., Weber A. (2013). Difference of interleukin-23 receptor gene haplotype variants in ulcerative colitis compared to Crohn’s disease and psoriasis. Inflamm. Res..

[71] Kara S., Pirela-Morillo G.A., Gilliam C.T., Wilson G.D. (2019). Identification of novel susceptibility genes associated with seven autoimmune disorders using whole genome molecular interaction networks. J. Autoimmun..

[72] Ryan D.G., O’Neill L.A.G. (2017). Krebs cycle rewired for macrophage and dendritic cell effector functions. FEBS Lett..

[73] Zasłona Z., O’Neill L.A.J. (2020). Cytokine-like Roles for Metabolites in Immunity. Mol. Cell.

[74] Sriram K., Insel P.A. (2018). G Protein-Coupled Receptors as Targets for Approved Drugs: How Many Targets and How Many Drugs?. Mol. Pharmacol..

[75] Sands W.A., Palmer T.M. (2008). Regulating gene transcription in response to cyclic AMP elevation. Cell Signal..

