# Supplementary material for: Apremilast Pharmacogenomics in Russian Patients with Moderate-to-Severe and Severe Psoriasis
Source: J Pers Med. 2020 Dec 29;11(1):20. doi: 10.3390/jpm11010020 (PMC7823747; doi:10.3390/jpm11010020)
Supplement: Supplementary file 1 [file jpm-11-00020-s001.pdf]

Supplementary Table 1. List of top SNP resulted from the association test of genome-wide pharmacogenomic study on apremilast between patients groups with distant therapy outcome (allele designation according Genome Reference Consortium Human Build 37 (GRCh37).

Chromosome number	SNP reference sequence	Chromosome position	Most frequent allele variant	Minimal frequency allele variant	Allele frequency in efficient target therapy response group	Allele frequency in the unsufficient target therapy response group	Coincidence probability
5	rs371208912	28107813	T	TATA	0	0,55	1,83E-06
2	rs12468345	59518670	G	A	0,75	0,2	6,38E-06
2	rs1344461	59544419	T	C	0,75	0,2	6,38E-06
2	rs10166053	59545031	G	A	0,75	0,2	6,38E-06
2	rs12478341	59546517	C	T	0,75	0,2	6,38E-06
2	rs11679165	59547991	G	A	0,75	0,2	6,38E-06
2	rs10182747	59519869	C	A	0,71	0,18	7,71E-06
2	rs11691103	59544147	C	T	0,71	0,18	7,71E-06
2	rs10192391	59545266	G	C	0,71	0,18	7,71E-06
2	rs13028418	59546095	C	T	0,71	0,18	7,71E-06
2	rs116436464	59555437	G	A	0,71	0,18	7,71E-06
23	rs35084576	2998500	C	T	0,68	0,04	7,99E-06
4	rs13124213	179875031	A	G	0	0,5	8,45E-06
4	rs34105287	179875449	G	GA	0	0,5	8,45E-06
4	rs4561967	179877696	A	G	0	0,5	8,45E-06
4	rs3943521	179877850	T	G	0	0,5	8,45E-06
4	rs1451428	179878094	C	A	0	0,5	8,45E-06
4	rs1903409	179878200	A	G	0	0,5	8,45E-06
4	rs2060771	179878367	C	T	0	0,5	8,45E-06
4	rs1839757	179878919	T	C	0	0,5	8,45E-06
4	rs6828097	179879147	C	T	0	0,5	8,45E-06
4	rs6851615	179879242	C	G	0	0,5	8,45E-06
4	rs6851276	179879278	T	C	0	0,5	8,45E-06
4	rs6851252	179879279	G	A	0	0,5	8,45E-06
4	rs10021175	179879485	A	C	0	0,5	8,45E-06

Supplementary Table 1. List of top SNP resulted from the association test of genome-wide pharmacogenomic study on apremilast between patients groups with distant therapy outcome (allele designation according Genome Reference Consortium Human Build 37 (GRCh37).

4	rs67655789	179879792	T	TATG	0	0,5	8,45E-06
4	rs10021825	179880039	A	G	0	0,5	8,45E-06
4	rs13122542	179880437	T	C	0	0,5	8,45E-06
4	rs6814835	179880495	T	A	0	0,5	8,45E-06
4	rs6841410	179880885	G	T	0	0,5	8,45E-06
4	rs28414971	179880948	T	C	0	0,5	8,45E-06
4	rs10024813	179881046	T	C	0	0,5	8,45E-06
4	rs1075183	179881743	G	T	0	0,5	8,45E-06
4	rs1075182	179881787	C	T	0	0,5	8,45E-06
4	rs28699520	179882372	A	G	0	0,5	8,45E-06
4	rs6830112	179883344	G	A	0	0,5	8,45E-06
4	rs6830337	179883428	G	A	0	0,5	8,45E-06
4	rs6844672	179885472	C	G	0	0,5	8,45E-06
4	rs141155679	179885969	T	TTTTC	0	0,5	8,45E-06
4	rs6850919	179886305	T	C	0	0,5	8,45E-06
4	rs12645034	179886711	C	T	0	0,5	8,45E-06
4	rs11131890	179887239	A	C	0	0,5	8,45E-06
4	rs6552320	179888435	G	A	0	0,5	8,45E-06
4	rs6552321	179888548	C	G	0	0,5	8,45E-06
4	rs28689131	179890992	T	C	0	0,5	8,45E-06
4	rs12108561	179891076	G	C	0	0,5	8,45E-06
4	rs62333826	179891529	G	A	0	0,5	8,45E-06
4	rs11131891	179892637	A	G	0	0,5	8,45E-06
4	rs17792161	179893411	G	C	0	0,5	8,45E-06
4	rs9992240	179895198	T	G	0	0,5	8,45E-06
4	rs4272057	179895526	G	A	0	0,5	8,45E-06
4	rs4423919	179895566	G	T	0	0,5	8,45E-06
4	rs13139913	179896260	C	T	0	0,5	8,45E-06
4	rs9998128	179896707	A	G	0	0,5	8,45E-06
4	rs28415348	179897406	C	T	0	0,5	8,45E-06

Supplementary Table 1. List of top SNP resulted from the association test of genome-wide pharmacogenomic study on apremilast between patients groups with distant therapy outcome (allele designation according Genome Reference Consortium Human Build 37 (GRCh37).

4	rs2060772	179898113	C	G	0	0,5	8,45E-06
4	rs13102434	179898167	C	T	0	0,5	8,45E-06
4	rs71601118	179901197	TTGTGTGTG	T	0	0,5	8,45E-06
4	rs34022518	179902400	T	C	0	0,5	8,45E-06
4	rs6812232	179903243	G	A	0	0,5	8,45E-06
4	rs150336386	179908853	C	T	0	0,5	8,45E-06
4	rs113143277	179909528	A	G	0	0,5	8,45E-06
4	rs141850912	179909745	A	G	0	0,5	8,45E-06
4	rs12645585	179912441	A	C	0	0,5	8,45E-06
4	rs35653024	179918367	A	G	0	0,5	8,45E-06
4	rs34462324	179918390	A	C	0	0,5	8,45E-06
5	rs2329722	28098757	G	A	0	0,5	8,45E-06
5	rs7712909	28109564	A	G	0	0,5	8,45E-06
5	rs35733073	28110318	C	CAT	0	0,5	8,45E-06
5	rs10067148	28112135	T	C	0	0,5	8,45E-06
5	rs6891780	28114207	T	C	0	0,5	8,45E-06
2	rs35032411	59545167	CA	C	0,68	0,15	8,63E-06
